# Time Trends of Ventricular Reconstruction and Outcomes among Patients with Left Ventricular Thrombus and Aneurysms

**DOI:** 10.3390/jcdd9120464

**Published:** 2022-12-15

**Authors:** Boqun Shi, Xieraili Tiemuerniyazi, Rui Zhang, Chenxi Song, Kongyong Cui, Dong Zhang, Lei Jia, Dong Yin, Hongjian Wang, Weihua Song, Wei Feng, Kefei Dou

**Affiliations:** 1 Cardiometabolic Medicine Center, Department of Cardiology, Fuwai Hospital, Chinese Academy of Medical Sciences & Peking Union Medical College/National Center for Cardiovascular Diseases, Beijing 100037, China; 2 Coronary Heart Disease Center, Department of Cardiology, Fuwai Hospital, Chinese Academy of Medical Sciences & Peking Union Medical College/National Center for Cardiovascular Diseases, Beijing 100037, China; 3 State Key Laboratory of Cardiovascular Disease, Beijing 100037, China; 4 Department of Cardiovascular Surgery, Fuwai Hospital, Chinese Academy of Medical Sciences & Peking Union Medical College/National Center for Cardiovascular Diseases, Beijing 100037, China

**Keywords:** surgical ventricular reconstruction, left ventricular thrombus, left ventricular aneurysm

## Abstract

**Background:** Clinical guidelines recommend surgical intervention when left ventricular thrombus (LVT) is complicated with left ventricular aneurysm (LVA). **Objectives:** This study aimed to review the changes in the treatment of LVT combined with LVA over the past 12 years at our center and to compare the efficacy of medical therapy and surgical treatment on patient outcomes. **Methods:** Between January 2009 and June 2021, 723 patients with LVT combined with LVA were enrolled, of whom 205 received surgical ventricular reconstruction (SVR) therapy and 518 received medical therapy. The following clinical outcomes were gathered via observation: all-cause death, cardiovascular death, and major adverse cardiovascular and cerebrovascular events (MACCEs; defined as the composite of cardiovascular death, ischemic stroke, and acute myocardial infarction). The median follow-up time was 1403 [707, 2402] days. **Results:** The proportion of SVR dropped yearly in this group of patients, from a peak of 64.5% in 2010 to 7.5% in 2021 (*p* for trend < 0.001). Meanwhile, the proportion of anticoagulant use increased quickly, from 8.0% in 2016 to 67.9% in 2021 (*p* for trend < 0.001). The incidence rates of all-cause mortality, cardiovascular death, and MACCEs were 12.9% (*n* = 93), 10.5% (*n* = 76), and 14.7% (*n* = 106), respectively. In the multivariable analysis, there were no significant differences in all-cause death (HR of 0.60, 95% CI of 0.32–1.13, *p* = 0.11), cardiovascular death (HR of 0.79, 95% CI of 0.41–1.50, *p* = 0.5), and MACCEs (HR of 0.82, 95% CI of 0.49–1.38, *p* = 0.5) between the two groups. The competing risk regression performed in the propensity score matching (PSM) and inverse probability of treatment weighting (IPTW) analyses was in line with the unmatched analysis. **Conclusions:** The rate of SVR dropped significantly among patients with both LVT and LVA, while there was an improvement in oral anticoagulant utilization. SVR with thrombus removal did not improve all-cause mortality and cardiovascular outcomes in patients with LVT and LVA. Ventricular aneurysm with thrombus may not be an indication for surgery.

## 1. Introduction

Left ventricular thrombus (LVT) is one of the severe complications of acute myocardial infarction (MI) and is proven to be associated with an increased risk of adverse events. In the era of revascularization, with the widespread application of percutaneous coronary intervention (PCI) and the advancement of medical therapy in the treatment of ventricular remodeling and thromboembolism, the incidence of LVT has been significantly reduced [1,2], decreasing gradually from 33% to approximately 10% [3].

LVT is not uncommon in patients with ischemic left ventricular aneurysm (LVA), and the most important treatment of LVT in these patients is the correction of its etiology. Therefore, coronary revascularization is the primary treatment for patients with LVT caused by ischemic heart disease. Meanwhile, the treatment of LVT itself is also crucial. Currently, treatment strategies for LVT include medical antithrombotic therapy after PCI and surgical thrombectomy concomitantly with coronary artery bypass grafting and surgical ventricular reconstruction (SVR). Anticoagulant therapy with a vitamin K antagonist is reasonable for patients with ST-segment elevation myocardial infarction and LVT [4,5], while more and more studies have been evaluating the efficacy of oral anticoagulants in this group of patients [6].

The European Society of Cardiology and European Association for Cardio-Thoracic Surgery (ESC/EACTS) guidelines on myocardial revascularization suggest considering left ventricular aneurysmectomy during coronary artery bypass grafting (CABG) in patients with NYHA Class III/IV, large LVA, or large thrombus formation, or if the aneurysm is the origin of arrhythmias (Class IIa, level C). SVR during CABG may be considered in selected patients treated in centers of expertise (Class IIb, level B) [7]. At the same time, they emphasize that the presence of LVT alone should not be considered an indication for open-heart surgery. Nowadays, there is an increase in usage of anticoagulation among patients discharged with LVT [8], while the proportion of LVA excision has decreased from 14.5% in 2006 to 9.8% in 2014 [9]. However, there are limited data on comparing the efficacy of medical and surgical treatment in patients with LVT and LVA, especially in Asian patients.

This retrospective study aimed to review the changes in the treatment of LVT combined with LVA over the past 12 years at our center and to compare the efficacy of medical therapy and surgical treatment on patient outcomes.

## 2. Methods

### 2.1. Study Design and Patient Population

**Figure 1:**
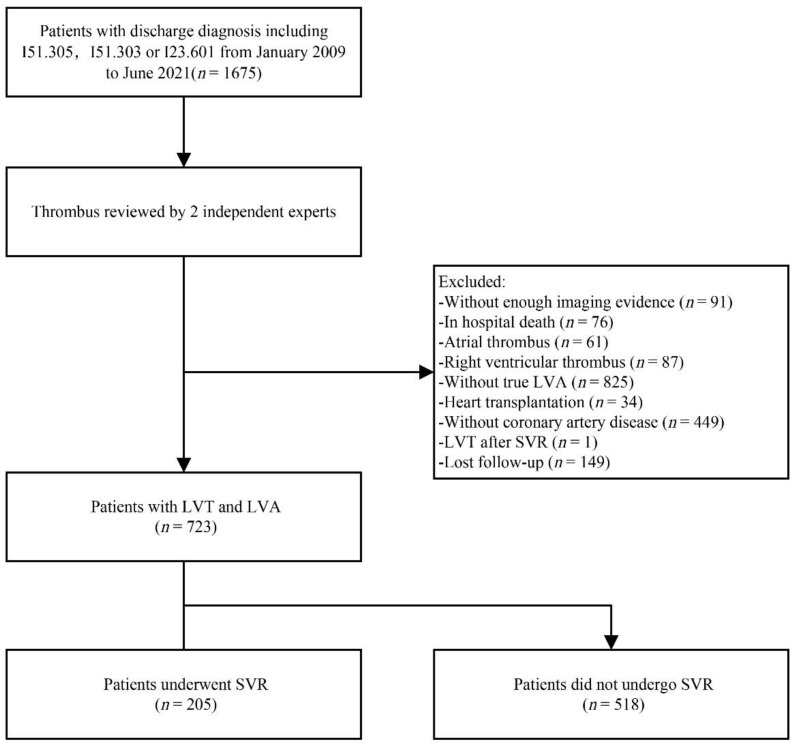
Study flow chart. LVA = left ventricular aneurysm. LVT = left ventricular thrombus. SVR = surgical ventricular reconstruction.

This study was a retrospective cohort study designed and reported according to the Strengthening the Reporting of Observational Studies in Epidemiology (STROBE) statement. The study protocol was conducted according to the Declaration of Helsinki. The Institutional Review Board of Fuwai Hospital approved the use of clinical data for this study. Written consent was waived owing to minimal patient risk. Oral consent was obtained at the time of the telephone interview. Among patients hospitalized at Fuwai Hospital Chinese Academy of Medical Sciences between January 2009 and June 2021, patients were determined to be eligible for inclusion following a search for the following terms and their ICD-10 (International Classification of Diseases 10th revision, for clinical use in Beijing) codes in the discharge diagnosis records in the electronic medical record system: “mural thrombosis of the left ventricle (I51.305)”, “ventricular thrombus (I51.303)”, and “mural thrombosis of the left ventricle after acute myocardial infarction (I23.601)”. Patients who met any of the following criteria were excluded: (1) patients without enough imaging evidence; (2) patients who suffered from in-hospital death; (3) patients with atrial or right ventricular thrombus; (4) patients without coronary artery disease and true LVA; (5) patients undergoing heart transplant during hospital stay; (6) patients diagnosed with LVT after SVR; (7) patients with incomplete follow-up data. The specific subject enrollment and screening process is illustrated in Figure 1. SVR techniques were described previously in [10]. Ultimately, this study included 723 patients. All participants were divided into two groups according to the treatment protocol: the SVR group (*n* = 205) and the non-SVR group (*n* = 518). Doctors decided to prefer SVR during coronary artery bypass grafting in patients with large LVA and large thrombus formation.

### 2.2. Baseline, Follow-Up, and Outcomes

Baseline data such as demographic features, past medical history, medications at discharge, and echocardiographic evaluation were retracted from electronic medical records. Follow-up was completed with regular outpatient visits and phone call interviews. The following clinical outcomes were gathered via observation: all-cause death, cardiovascular death, and major adverse cardiovascular and cerebrovascular events (MACCEs; defined as the composite of cardiovascular death, ischemic stroke, and acute myocardial infarction).

### 2.3. Left Ventricular Thrombus and Aneurysm Evaluation

This study assessed LVT and LVA using transthoracic echocardiography, contrast-enhanced CT, or cardiac magnetic resonance imaging. All patients underwent echocardiography at least once during hospitalization. A thin-walled bulging left ventricular shape that occurs during diastole and systole is an aneurysm. Only those with post-MI LVA were chosen for the study. Ischemic LVA was deemed certain when MI had already been diagnosed or at the time of LVA diagnosis. Hypokinesia or akinesia in segments next to the LVA without global hypokinesia was regarded as diagnostic of probable ischemic origin if the prior MI could not be determined. LVT shows as an echo-dense mass with borders different from the endocardium and is frequently adjacent to a segment contracting unnaturally. The attending physicians chose whether to perform cardiac magnetic resonance imaging or contrast-enhanced CT. Two experienced cardiologists independently interpreted the imaging data. They determined the location, shape, activity, and whether the LVT was multiple or calcified. A round LVT is defined as a thrombus with a protruding element that is not entirely attached to the ventricle wall. Left ventricular characteristics were also collected, including left ventricular end-diastolic dimension (LVEDD) and LVEF.

### 2.4. Other Covariates

The estimated glomerular filtration rate (eGFR) was calculated according to the CKD-EPI (Chronic Kidney Disease Epidemiology Collaboration) creatine equation (2021) [11]. Diabetes was diagnosed in patients presenting fasting plasma glucose ≥ 7.0 mmol/L, two-hour plasma glucose of the oral glucose tolerance test ≥ 11.1 mmol/L, hemoglobin A1c (HbA1c) ≥ 6.5% at baseline, or current use of hypoglycemic drugs or insulin [12]. Hypertension was defined as a history of hypertension, currently taking antihypertensive drugs, or recorded systolic blood pressure ≥ 140 mmHg or diastolic blood pressure ≥ 90 mmHg three or more consecutive times.

### 2.5. Statistical Analysis

All continuous variables in our study were expressed using medians with their 25th and 75th percentiles. Continuous variables were compared with Mann-Whitney-U test. Categorical variables were presented as numbers (%) and were compared using the χ^2^ test or Fisher’s exact test. A *p*-value of <0.05 was considered statistically significant. All statistical analyses were performed using R 4.2.1 (R Core Team, Vienna, Austria).

Cumulative incidence functions (CIFs) were generated for the competing risks of death during the follow-up period. The competing risk regression model was fit using the cmprsk package in R. Comparisons were made between groups using Gray’s test. Time-to-event curves were illustrated using the Nelson–Aalen method. We used a multivariable competing risk regression model to estimate hazard ratios and confidence intervals, with SVR as the dependent variable and the following factors as covariates: age, gender, eGFR < 60 mL/min/1.73 m^2^, body mass index, LVEF ≤ 40%, diabetes mellitus, prior stroke, and atrial fibrillation. These variables were chosen because they were related to clinical outcomes in the univariate analysis (*p* < 0.10) or were available at baseline, which is known to be relevant to outcomes in patients with coronary artery disease.

We used propensity score matching (PSM) with the best approach and a 1:1 ratio to balance the baseline confounding factors between the two groups. Multivariable logistic regression was used to identify the propensity score estimation. The Supplementary Materials define the model’s variables in detail. To assess the effectiveness of PSM in reducing the baseline disparity between the two groups, the standardized mean difference (SMD) was utilized. An SMD of 10% or lower suggested a productive PSM. In order to establish a weighted cohort that could balance the covariate bias and assess the outcomes’ robustness, we also used inverse probability of treatment weighting (IPTW).

## 3. Results

### 3.1. Characteristics of the Patients

A total of 723 patients were enrolled, 205 receiving SVR therapy and 518 receiving medical therapy. Table 1 lists the baseline features of the individuals with ischemic LVA and LVT. The population had a median age of 58.0 [51.0, 66.0] years, and most were male (88.0%). More than half of the patients (55.2%) had hypertension, while 39.0% were complicated with diabetes mellitus. Regarding prescriptions at discharge, 391 (54.1%) patients received antiplatelet therapy only, and 48 (6.6%) received anticoagulation therapy only, while the remainders were treated with the combination of antiplatelet and anticoagulation therapy. Reduced LVEF (LVEF ≤ 40%) was common in nearly half of the population (47.6%). LVT was predominantly located at apical segments (93.6%, *n* = 677). Round LVT was detected in 400 (55.3%) patients, and calcified LVT was observed in 88 (12.2%).

In the SVR group, 144 (70.2%) patients underwent linear ventriculoplasty, while 61 (29.8%) received non-linear repair, including modified left ventricular reconstruction and endocardial patch reconstruction. Concomitant ventricular thrombectomy was performed in 196 (95.6%) of the patients. Compared with patients without SVR, those who underwent SVR were younger (58.0 [50.0, 63.0] years vs. 59.0 [51.0, 67.0] years, *p* = 0.049) and had a lower proportion of comorbidities such as hypertension (46.8% vs. 58.5%, *p* = 0.006) and atrial fibrillation (2.0% vs. 9.3%, *p* = 0.001). Antithrombotic medication at discharge differed significantly between the two groups. More patients in the SVR group were prescribed aspirin (93.7% vs. 76.1%, *p* < 0.001), while a lower proportion of them received clopidogrel (30.7% vs. 65.8%, *p* < 0.001). The majority of patients who underwent SVR received antiplatelet therapy only (64.4% vs. 50.0%, *p* = 0.001) or aspirin with anticoagulants (10.8% vs. 24.9%, *p* < 0.001), and few of them received DOAC therapy (19.7% vs. 0.5%, *p* < 0.001). As for the echocardiographic evaluation, patients in the SVR group shared slightly higher LVEF (42.0% vs. 40.7%, *p* = 0.026), while the LVEDD was comparable. In terms of LVT and LVA morphology, the SVR group had larger LVT (26.5 vs. 24.0 mm, *p* = 0.012) and LVA (41.0 vs. 39.0 mm, *p* = 0.002) (Table 1).

The PSM analysis achieved 205 pairs of matched patients, while the IPTW analysis resulted in 716.24 patients (516.34 without SVR and 199.90 with SVR). Baseline characteristics were considered to be well balanced both in the PSM and IPTW analyses (SMD < 0.100 and *p* < 0.05 for all of the variables enrolled) (Tables S1 and S2, and Figure S1).

### 3.2. Time Trends of SVR and Anticoagulation Therapy

**Figure 2:**
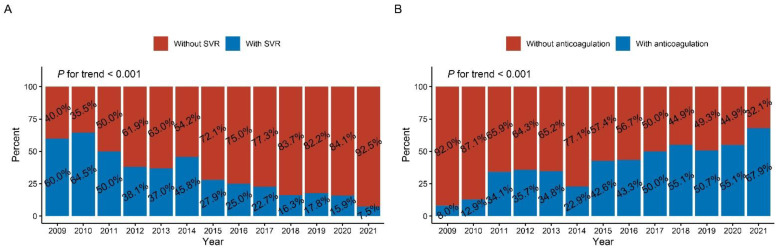
Time trends of therapy among patients with LVT and LVA from 2009 to 2021: (**A**) SVR; (**B**) anticoagulation therapy. LVT = left ventricular thrombus. LVA = left ventricular aneurysm. SVR = surgical ventricular reconstruction.

The proportion of SVR dropped yearly in this group of patients, from a peak of 64.5% in 2010 to 7.5% in 2021 (*p* for trend < 0.001). Meanwhile, the proportion of anticoagulant use increased quickly (Figure 2B), from 8.0% in 2009 to 67.9% in 2021 (*p* for trend < 0.001) (Figure 2).

### 3.3. Competing Risks of Adverse Outcomes

**Figure 3:**
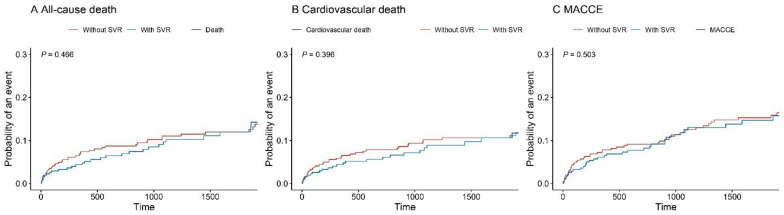
Time-to-event curves according to SVR group. CR = competing risk. MACCEs = major adverse cardiac and cerebrovascular events. (**A**) All-cause death; (**B**) Cardiovascular death; (**C**) MACCE.

Follow-up was completed by 91.1%, and the median follow-up time was 1403 [707, 2402] days. The incidence rates of all-cause mortality, cardiovascular death, and MACCEs were 12.9% (*n* = 93), 10.5% (*n* = 76) and 14.7% (*n* = 106), respectively. As shown in Table 2, patients without SVR were more likely to suffer from all-cause death (15.1% vs. 7.3%), cardiovascular death (12.0% vs. 6.8%), and MACCEs (16.4% vs. 10.2%) when than those who received SVR. However, time-to-event curves demonstrated that adverse outcomes were similar (Gray’s test, all *p* > 0.05) (Figure 3). The univariable competing risk regression analysis showed that SVR significantly reduced all-cause mortality (HR of 0.55, 95% CI of 0.32–0.97, *p* = 0.038). In the multivariable analysis, however, there were no significant differences in all-cause death (HR of 0.60, 95% CI of 0.32–1.13, *p* = 0.11), cardiovascular death (HR of 0.79, 95% CI of 0.41–1.50, *p* = 0.5), and MACCEs (HR of 0.82, 95% CI of 0.49–1.38, *p* = 0.5) between the two groups. To evaluate the robustness of the outcomes, competing risk regression was also performed in the PSM and IPTW analyses. The results showed that SVR was not independently associated with all-cause death (PSM, HR of 0.77, 95% CI of 0.40–1.50, *p* = 0.5; IPTW, HR of 0.71, 95% CI of 0.37–1.35, *p* = 0.292), cardiovascular death (PSM, HR of 0.93, 95% CI of 0.46–1.88, *p* = 0.8; IPTW, HR of 0.88, 95% CI of 0.45–1.71, *p* = 0.702), and MACCEs (PSM, HR of 1.06, 95% CI of 0.59–1.92, *p* = 0.8; IPTW, HR of 0.92, 95% CI of 0.54–1.57, *p* = 0.751), which was in line with the unmatched analysis.

**Figure 4:**
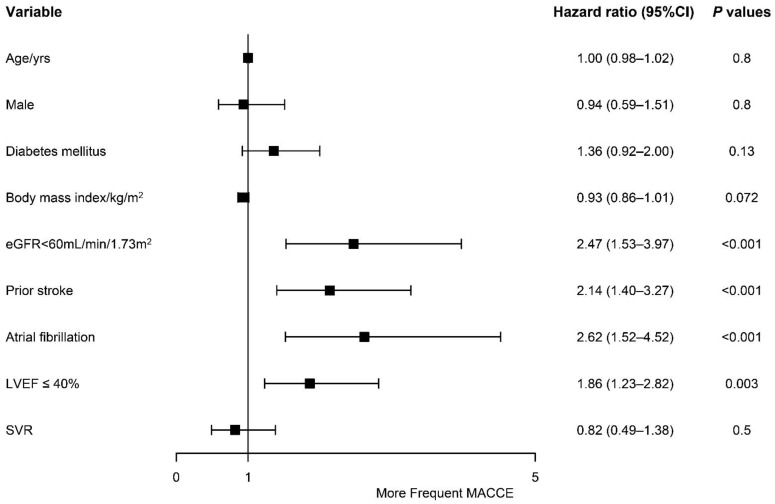
Association between baseline characters and the risk of major adverse cardiac and cerebrovascular events according to a multivariable analysis. CI = confidence interval. eGFR = estimated glomerular filtration rate. LVEF = left ventricular ejection fraction. SVR = Surgical ventricular reconstruction.

A multivariate analysis was also performed to explore the potential risk factors for the adverse events, which showed that eGFR < 60 mL/min/1.73 m^2^ (HR of 2.47, 95% CI of 1.53–3.97, *p* < 0.001), prior stroke (HR of 2.14, 95% CI of 1.40–3.27, *p* < 0.001), atrial fibrillation (HR of 2.62, 95% CI of 1.52–4.52, *p* < 0.001), and LVEF ≤ 40% (HR of 1.86, 95% CI of 1.23–2.82, *p* = 0.003) were independently associated with an increased risk of MACCEs (Figure 4).

Of 196 patients receiving LVT removal, 32.1% (*n* = 63) were prescribed oral anticoagulation therapy. The multivariable analysis demonstrated that oral anticoagulation therapy was not associated with all-cause death (HR of 0.64, 95% CI of 0.16–2.57, *p* = 0.5), cardiovascular death (HR of 0.64, 95% CI of 0.16–2.57, *p* = 0.5), or MACCEs (HR of 0.36, 95% CI of 0.09–1.38, *p* = 0.13).

## 4. Discussion

In this retrospective cohort study, we compared the clinical outcomes of patients with ischemic LVA followed by LVT who received medical therapy with or without SVR. Our key findings are as follows: (i) There was a decrease in the usage of SVR among patients with ischemic LVA and LVT during the last decades at our center, while the opposite was observed regarding the usage of oral anticoagulants; there was a tremendous improvement in oral anticoagulant use. (ii) SVR showed limited benefit on the patient outcomes, which did not show statistical difference. To our knowledge, our study involved the largest LVT cohort worldwide.

### 4.1. Trends in the Incidence of LVT

Left ventricular thrombus is one of the major complications of acute myocardial infarction, followed by the formation of LVA. Previously, researchers reported that the incidence of LVT after acute myocardial infarction could be as high as 33% [13], compromising the patient outcomes. Fortunately, this number has dropped rapidly with the emergence of thrombolytic [14], antithrombotic, and primary PCI therapy. Osherov et al. [15] reported that the rate of LVT is 6.2% in patients with anterior wall acute myocardial infarction after PCI. Later, in their prospective multicenter study, Meurin et al. [16] observed a 26% incidence of LVT after anterior wall acute myocardial infarction. However, this study enrolled patients with low LVEF (<45%), which is confirmed to be an independent risk factor for LVT [15,17]. Recent meta-analyses showed that the occurrence of LVT after PCI is 4–6%. At the same time, it is still as high as 10.0% in overall anterior wall acute myocardial infarction patients and 19.2% in those with LVEF < 50% [18,19]. During the last decade, the incidence of LVT declined at our center, ranging from 0.05% to 0.18% among discharged patients [20].

### 4.2. Impact of LVT on Prognosis and Treatment

The most severe consequence of LVT is an increased risk of systemic embolism [21]. Earlier, in 1993, Vaitkus et al. [22] noticed that the incidence of systemic embolism increased approximately about 9% in their meta-analysis. In a more recent study, researchers reported a 16% incidence of systemic embolism after myocardial infarction [23]. This study also demonstrated a significant risk reduction with the administration of anticoagulants. Thus, it is recommended that anticoagulation therapy is started immediately [5,24].

Meanwhile, surgical thrombectomy is another option [25]. In this study, we observed that there was a decrease in the usage of surgical treatment, while the opposite was observed regarding oral anticoagulation therapy. Similar results were reported by Bhardwaj et al. in America [9]. They noticed that the overall percentage of LVA excision decreased from 14.5% in 2006 to 9.8% in 2014 (*p* for trend = 0.003). This might be attributed to the decrease in the incidence of LVT and the development of anticoagulation therapy. Unfortunately, however, there is a paucity of data for comparing the patient outcomes after medical therapy and surgical treatment.

### 4.3. Comparison of Medical and Surgical Treatment

Clinical guidelines recommend SVR, specifically left ventricular aneurysmectomy, during coronary artery bypass grafting in patients with large LVA and LVT [7]. However, with the development of antithrombotic therapy, especially anticoagulation therapy, more and more patients with LVA and LVT have been receiving medical treatment adjunctively to primary PCI, and it is not recommended to perform open-heart surgery where LVT is the only indication [26]. Limited studies exist regarding the comparison of medical and surgical treatments. Lee et al. [27] reported that the incidence of thromboembolic events after anticoagulation therapy was higher (17%) than after surgical treatment but without statistical difference between the groups. Nevertheless, their study was restricted to a small sample size, including patients with LVT caused by non-ischemic etiology. In this study, we compared the clinical outcomes, including mortality and MACCEs, rather than thromboembolic events. We noticed that all-cause death and cardiovascular death after medical treatment were almost twice as frequent as those after surgical treatment, and the occurrence of MACCEs was also 1.6 times that of the surgical treatment during the follow-up period. Although the unmatched analysis showed statistical significance, this difference disappeared in the PSM and IPTW analyses. Our results were consistent with those of prior studies. The possible explanations might be as follows: (i) With the advancement of reperfusion and anticoagulation therapy, LVA was diagnosed in the early stage, and anticoagulants were used immediately. (ii) Although approaches such as PSM and IPTW were used to deal with baseline imbalances, differences existed regarding the indications for primary PCI followed by medical treatment and surgical treatment with thrombectomy. (iii) Less patients underwent surgical treatment, with the latter including linear ventriculoplasty, modified left ventricular reconstruction, and endocardial patch reconstruction, and about a half of the patients received antiplatelet therapy without anticoagulants after the surgery. (iv) The follow-up time was relatively short in this study.

### 4.4. Whether Postoperative Anticoagulant Therapy Is Required

Currently, research on treating left ventricular thrombosis focuses on anticoagulation therapy [28,29], especially the comparison between DOAC and warfarin [30]. However, it is not clear whether anticoagulation therapy is necessary after LVT removal. According to our results, postoperative oral anticoagulation therapy does not benefit patients.

### 4.5. Risk Factors for the Prognosis

LVT is a marker of increased risk of a long-term adverse event, which may persist even after resolution [25]. That is to say, patients with LVT are at a higher risk. Therefore, risk factors for the formation of LVT might also influence the clinical outcome of these patients. Previous studies concluded that anterior ST-segment elevation myocardial infarction, left anterior descending-related infarct, left ventricular wall motion abnormalities, and lower LVEF are the potential predictors of LVT [17,19]. Elsewhere, researchers [27] reported that dilated cardiomyopathy, a history of stroke, female gender, and LVEDD are the potential risk factors for adverse events. In this study, we noticed that renal dysfunction (which was defined as eGFR < 60 mL/min/1.73 m^2^), a history of stroke, atrial fibrillation, and reduced LVEF (≤40%) were potential predictors of MACCEs. Therefore, patients with these risk factors should be evaluated carefully.

This study was a retrospective observational analysis involving the largest single-center cohort of LVT patients. Our study is also the first to summarize trends in SVR in patients with LVT and LVA over ten years and to compare the benefits of surgical versus conservative medical treatment. There were differences in baseline characteristics between those with SVR and without SVR. However, the results persisted in the multivariate analysis and sensitivity analysis. SVR did not benefit patients with LVT and LVA in terms of all-cause mortality and cardiovascular outcomes. The failure to significantly improve the prognosis may account for the decline in the practice of SVR.

## 5. Limitations

Firstly, this study was limited by its retrospective nature, particularly the possibility of unmeasured confounders accounting for the difference in adverse events between groups. Secondly, our study included patients with LVT diagnosed using various imaging studies, and not all patients received cardiac magnetic resonance imaging. This may have caused heterogeneity issues. Thirdly, the follow-up of bleeding events was not available in our study. So, we do not know whether SVR can significantly reduce major bleeding events.

## 6. Conclusions

In this retrospective study, we discussed the time trends of SVR and anticoagulation therapy among Asian patients with LVT and LVA. The rate of SVR dropped significantly among patients with both LVT and LVA, while there was an improvement in oral anticoagulant utilization. SVR with thrombus removal did not improve all-cause mortality and cardiovascular outcomes for patients with LVT and LVA. Ventricular aneurysm with thrombus may not be an indication for surgery.

## Figures and Tables

**Table 1:** Baseline features in the whole population and according to SVR group.

	Overall	Non-SVR Group	SVR Group	*p*-Value	SMD
*n*	723	518	205		
Demographics					
Age (years), median [Q1, Q3]	58.0 [51.0, 66.0]	59.0 [51.0, 67.0]	58.0 [50.0, 63.0]	0.049	0.156
Male, n (%)	636 (88.0)	451 (87.1)	185 (90.2)	0.29	0.1
Body mass index (kg/m^2^), median [Q1, Q3]	25.1 [23.2, 27.3]	25.2 [23.2, 27.4]	24.9 [23.4, 27.3]	0.687	0.046
Past medical history					
Hypertension, n (%)	399 (55.2)	303 (58.5)	96 (46.8)	0.006	0.235
Diabetes mellitus, n (%)	282 (39.0)	214 (41.3)	68 (33.2)	0.053	0.169
eGFR < 60 mL/min/1.73 m^2^, n (%)	113 (15.6)	83 (16.0)	30 (14.6)	0.726	0.039
Stroke, n (%)	118 (16.3)	90 (17.4)	28 (13.7)	0.268	0.103
CABG, n (%)	17 (2.4)	17 (3.3)	0	0.019	0.261
PCI, n (%)	160 (22.1)	120 (23.2)	40 (19.5)	0.333	0.089
Atrial fibrillation, n (%)	52 (7.2)	48 (9.3)	4 (2.0)	0.001	0.322
Antithrombotic medications					
Aspirin, n (%)	586 (81.1)	394 (76.1)	192 (93.7)	<0.001	0.506
Clopidogrel, n (%)	404 (55.9)	341 (65.8)	63 (30.7)	<0.001	0.75
Ticagrelor, n (%)	35 (4.8)	27 (5.2)	8 (3.9)	0.584	0.063
P2Y12 inhibitor, n (%)	439 (60.7)	368 (71.0)	71 (34.6)	<0.001	0.783
DAPT, n (%)	366 (50.6)	296 (57.1)	70 (34.1)	<0.001	0.475
VKA, n (%)	213 (29.5)	150 (29.0)	63 (30.7)	0.703	0.039
DOAC, n (%)	103 (14.2)	102 (19.7)	1 (0.5)	<0.001	0.673
Antiplatelet therapy only, n (%)	391 (54.1)	259 (50.0)	132 (64.4)	0.001	0.294
Anticoagulation therapy only, n (%)	48 (6.6)	45 (8.7)	3 (1.5)	0.001	0.334
Aspirin with anticoagulants, n (%)	107 (14.8)	56 (10.8)	51 (24.9)	<0.001	0.374
Clopidogrel with anticoagulants, n (%)	66 (9.1)	66 (12.7)	0	<0.001	0.54
DAPT with anticoagulants	93 (12.9)	84 (16.2)	9 (4.4)	<0.001	0.397
Echocardiographic evaluation					
LVEDD (mm), median [Q1, Q3]	56.0 [51.0, 60.9]	56.0 [52.0, 60.6]	55.1 [51.0, 61.0]	0.231	0.131
LVEF (%), median [Q1, Q3]	42.0 [36.0, 48.0]	40.7 [35.0, 48.0]	42.0 [38.0, 50.0]	0.026	0.186
LVEF ≤ 40%, n (%)	344 (47.6)	258 (49.8)	86 (42.0)	0.068	0.158
Apical LVT, n (%)	677 (93.6)	490 (94.6)	187 (91.2)	0.132	0.132
Round LVT, n (%)	400 (55.3)	299 (57.7)	101 (49.3)	0.048	0.17
Mobile LVT, n (%)	28 (3.9)	21 (4.1)	7 (3.4)	0.851	0.034
Multiple LVT, n (%)	32 (4.4)	27 (5.2)	5 (2.4)	0.152	0.145
Calcified LVT, n (%)	88 (12.2)	68 (13.1)	20 (9.8)	0.261	0.106
LVT largest diameter (mm), median [Q1, Q3]	25.9 [18.0, 34.0]	24.0 [17.3, 33.0]	26.53 [19.0, 35.0]	0.012	0.128
LVA largest diameter (mm), median [Q1, Q3]	40.0 [32.0, 46.0]	39.0 [32.0, 45.0]	41.0 [34.0, 49.0]	0.002	0.254

SVR = surgical ventricular reconstruction. eGFR = estimated glomerular filtration rate. CABG = coronary artery bypass grafting. PCI = percutaneous coronary intervention. DAPT = dual antiplatelet therapy. VKA = vitamin-K antagonist. DOAC = direct oral anticoagulant. LVEDD = left ventricular end diastolic dimension. LVEF = left ventricular ejection fraction. LVT = left ventricular thrombus. LVA = left ventricular aneurysm.

**Table 2:** Outcomes in the whole cohort and according to SVR group.

			Univariable Analysis		Multivariable Analysis		PSM Analysis		IPTW Analysis	
	Without SVR (*n* = 518)	With SVR (*n* = 205)	Hazard Ratio (95% CI)	*p*-Value	Hazard Ratio (95% CI)	*p*-Value	Hazard Ratio (95% CI)	*p*-Value	Hazard Ratio (95% CI)	*p*-Value
All-cause death	78 (15.1%)	15 (7.3%)	0.55 (0.32–0.97)	0.038	0.60 (0.32–1.13)	0.110	0.77 (0.40–1.50)	0.500	0.71 (0.37–1.35)	0.292
Cardiovascular death	62 (12.0%)	14 (6.8%)	0.69 (0.38–1.23)	0.200	0.79 (0.41–1.50)	0.500	0.93 (0.46–1.88)	0.800	0.88 (0.45–1.71)	0.702
MACCEs	85 (16.4%)	21 (10.2%)	0.75 (0.46–1.20)	0.200	0.82 (0.49–1.38)	0.500	1.06 (0.59–1.92)	0.800	0.92 (0.54–1.57)	0.751

Values are *n* (%). SVR = surgical ventricular reconstruction. MACCEs = major adverse cardiovascular and cerebrovascular events. CI = confidence interval. PSM = propensity score matching. IPTW = inverse probability of treatment weighting.

## Data Availability

The data presented in this study are available on request from the corresponding author. The data are not publicly available due to privacy and ethical restrictions.

## Supplementary Materials

The following supporting information can be downloaded at: https://www.mdpi.com/article/10.3390/jcdd9120464/s1. Table S1. Baseline features according to SVR group after propensity score matching. Table S2 Baseline features according to SVR group after inverse probability of treatment weighting. Figure S1 Standardized mean difference (SMD) between the 2 groups before and after propensity score matching and propensity score weight.

## Abbreviations

LVT = left ventricular thrombus. MI = myocardial infarction. PCI = percutaneous coronary intervention. ESC/EACTS = European Society of Cardiology and European Association for Cardio-Thoracic Surgery. CABG = coronary artery bypass grafting. SVR = surgical ventricular reconstruction. LVA = left ventricular aneurysm. MACCEs = major adverse cardiac and cerebrovascular events. LVEDD = left ventricular end diastolic dimension. LVEF = left ventricular ejection fraction. eGFR = estimated glomerular filtration rate. PSM = propensity score matching. SMD = standardized mean difference. IPTW = inverse probability of treatment weighting. DOAC = direct oral anticoagulant.
