# Supplementary material for: Time Trends of Ventricular Reconstruction and Outcomes among Patients with Left Ventricular Thrombus and Aneurysms
Source: J Cardiovasc Dev Dis. 2022 Dec 15;9(12):464. doi: 10.3390/jcdd9120464 (PMC9784406; doi:10.3390/jcdd9120464)
Supplement: Supplementary file 1 [file jcdd-09-00464-s001.zip › jcdd-2031137-supplementary.docx]

**SUPPLEMENTAL MATERIALS**

**1.Supplemental Methods**

The variables included in the model estimating propensity score: age, gender, body mass index, hypertension, diabetes mellitus, eGFR<60ml/min/1.73m^2^, prior stroke, prior CABG, prior PCI, atrial fibrillation, LVEDD, LVEF, apical LVT, round LVT, mobile LVT, multiple LVT, calcified LVT, LVT largest diameter and LVA largest diameter.

2.Supplemental Tables

Table S1. Baseline features according to SVR group after propensity score matching.

|  | **Without SVR** | **With SVR** | **P value** | **SMD** |
| --- | --- | --- | --- | --- |
| n | 205 | 205 |  |  |
| Demographic |  |  |  |  |
| Age | 56.00 [49.00, 65.00] | 58.00 [50.00, 63.00] | 0.725 | 0.034 |
| Male | 188 (91.7) | 185 (90.2) | 0.73 | 0.051 |
| Body mass index/kg/m^2^ | 25.06 [23.53, 27.14] | 24.92 [23.36, 27.27] | 0.653 | 0.069 |
| Past medical history |  |  |  |  |
| Hypertension | 101 (49.3) | 96 (46.8) | 0.693 | 0.049 |
| Diabetes mellitus | 73 (35.6) | 68 (33.2) | 0.677 | 0.051 |
| eGFR<60 ml/min/1.73m^2^ | 28 (13.7) | 30 (14.6) | 0.887 | 0.028 |
| Prior stroke | 28 (13.7) | 28 (13.7) | >0.99 | <0.001 |
| Prior CABG | 0 (0.0) | 0 (0.0) | NA | <0.001 |
| Prior PCI | 40 (19.5) | 40 (19.5) | >0.99 | <0.001 |
| Atrial fibrillation | 5 (2.4) | 4 (2.0) | >0.99 | 0.033 |
| Imageological examination |  |  |  |  |
| LVEDD | 55.00 [51.00, 60.00] | 55.08 [51.00, 61.00] | 0.987 | 0.047 |
| LVEF | 45.00 [38.00, 49.00] | 42.00 [38.00, 50.00] | 0.473 | 0.06 |
| LVEF<=40% | 79 (38.5) | 86 (42.0) | 0.546 | 0.07 |
| Apical LVT | 187 (91.2) | 187 (91.2) | 1 | <0.001 |
| Round LVT | 111 (54.1) | 101 (49.3) | 0.374 | 0.098 |
| Mobile LVT | 8 (3.9) | 7 (3.4) | 1 | 0.026 |
| Multiple LVT | 6 (2.9) | 5 (2.4) | 1 | 0.03 |
| Calcified LVT | 19 (9.3) | 20 (9.8) | 1 | 0.017 |
| LVT largest diameter/mm | 25.88 [18.00, 34.00] | 26.53 [19.00, 35.00] | 0.198 | 0.046 |
| LVA largest diameter/mm | 41.00 [34.00, 46.00] | 41.00 [34.00, 49.00] | 0.35 | 0.082 |

Data are median (IQR). SVR=Surgical ventricular reconstruction. eGFR=estimated glomerular filtration rate. CABG=coronary artery bypass grafting. PCI=percutaneous coronary intervention. LVEDD=left ventricular end diastolic dimension. LVEF=left ventricular ejection fraction. LVT=left ventricular thrombus. LVA=left ventricular aneurysm.

Table S2 Baseline features according to SVR group after inverse probability of treatment weighting.

|  | **Without SVR** | **With SVR** | **P value** | **SMD** |
| --- | --- | --- | --- | --- |
| n | 516.34 | 199.90 |  |  |
| Demographic |  |  |  |  |
| Age | 58.00 [50.02, 67.00] | 58.59 [51.00, 63.00] | 0.362 | 0.065 |
| Male | 454.7 (88.1) | 177.6 (88.9) | 0.785 | 0.025 |
| Body mass index/kg/m^2^ | 25.14 [23.18, 27.34] | 25.03 [23.51, 27.55] | 0.608 | 0.038 |
| Past medical history |  |  |  |  |
| Hypertension | 286.0 (55.4) | 112.0 (56.0) | 0.884 | 0.013 |
| Diabetes mellitus | 201.9 (39.1) | 74.1 (37.1) | 0.652 | 0.042 |
| eGFR<60 ml/min/1.73m^2^ | 77.8 (15.1) | 24.2 (12.1) | 0.309 | 0.086 |
| Prior stroke | 84.6 (16.4) | 31.9 (16.0) | 0.904 | 0.012 |
| Prior CABG | 12.2 (2.4) | 0.0 (0.0) | 0.011 | 0.22 |
| Prior PCI | 115.5 (22.4) | 43.3 (21.7) | 0.854 | 0.017 |
| Atrial fibrillation | 37.1 (7.2) | 9.8 (4.9) | 0.468 | 0.096 |
| Imageological examination |  |  |  |  |
| LVEDD | 56.00 [51.00, 60.00] | 56.00 [52.00, 62.00] | 0.587 | 0.023 |
| LVEF | 41.00 [36.00, 48.00] | 41.00 [37.00, 47.00] | 0.883 | 0.016 |
| LVEF<=40% | 247.3 (47.9) | 95.1 (47.6) | 0.942 | 0.007 |
| Apical LVT | 484.2 (93.8) | 188.3 (94.2) | 0.819 | 0.018 |
| Round LVT | 285.7 (55.3) | 107.7 (53.9) | 0.753 | 0.029 |
| Mobile LVT | 20.3 (3.9) | 6.4 (3.2) | 0.641 | 0.04 |
| Multiple LVT | 22.8 (4.4) | 8.9 (4.5) | 0.984 | 0.002 |
| Calcified LVT | 62.0 (12.0) | 22.1 (11.0) | 0.744 | 0.03 |
| LVT largest diameter/mm | 25.00 [18.00, 34.00] | 26.53 [19.00, 35.00] | 0.094 | 0.072 |
| LVA largest diameter/mm | 40.00 [32.00, 46.00] | 39.87 [33.00, 47.00] | 0.702 | 0.019 |

Data are median (IQR). SVR=Surgical ventricular reconstruction. eGFR=estimated glomerular filtration rate. CABG=coronary artery bypass grafting. PCI=percutaneous coronary intervention. LVEDD=left ventricular end diastolic dimension. LVEF=left ventricular ejection fraction. LVT=left ventricular thrombus. LVA=left ventricular aneurysm.

**3.Supplemental Figures**


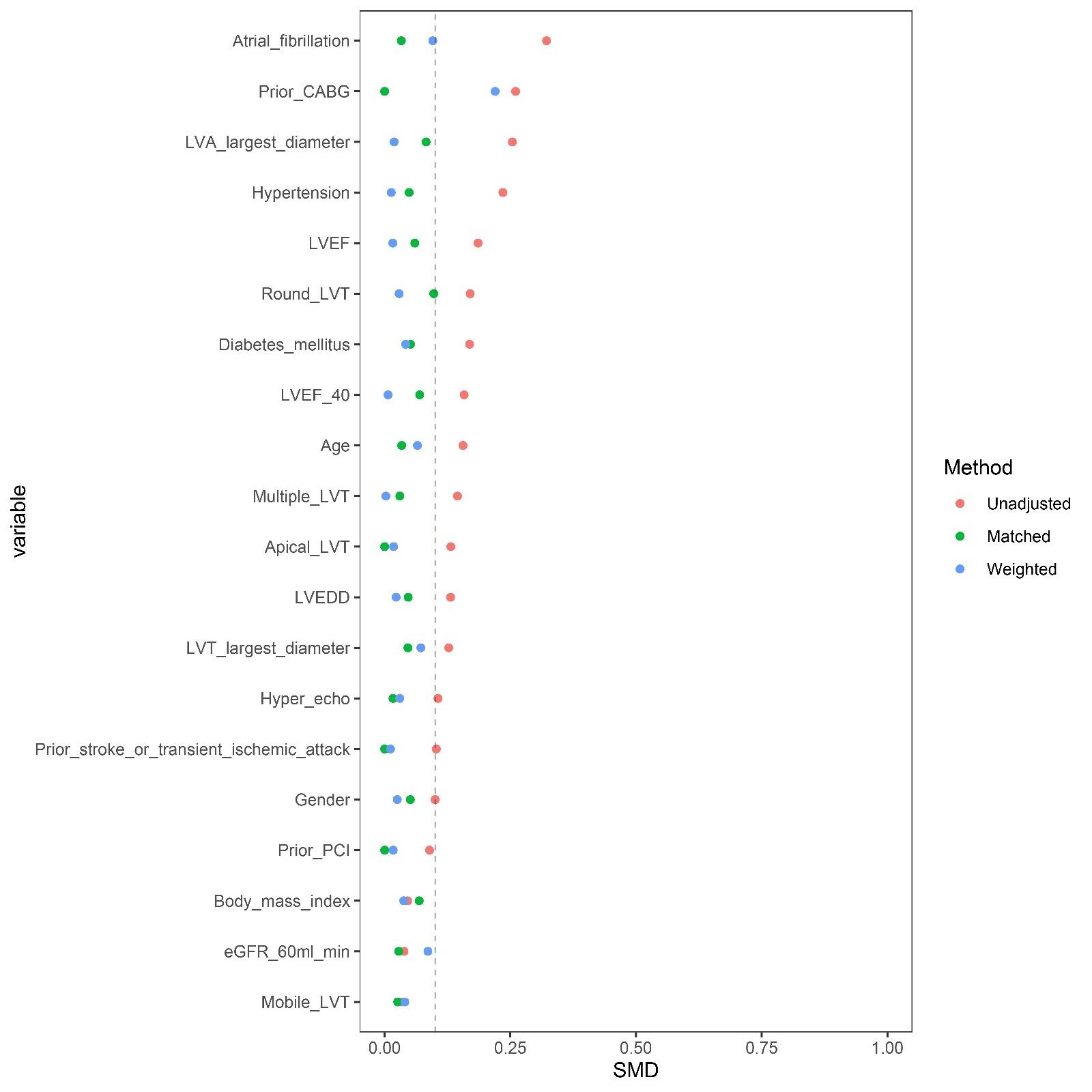
 **Figure S1 Standardized mean difference (SMD) between the 2 groups before and after propensity score matching and propensity score weight.**

CABG=coronary artery bypass grafting. LVA= left ventricular aneurysm. LVEF=left ventricular ejection fraction. LVT=left ventricular thrombus. LVEDD=left ventricular end diastolic dimension. PCI=percutaneous coronary intervention. eGFR=estimated glomerular filtration rate.
